# Regulation of IL-10 and IL-12 production and function in macrophages and dendritic cells

**DOI:** 10.12688/f1000research.7010.1

**Published:** 2015-12-17

**Authors:** Xiaojing Ma, Wenjun Yan, Hua Zheng, Qinglin Du, Lixing Zhang, Yi Ban, Na Li, Fang Wei

**Affiliations:** 1 State Key Laboratory of Microbial Metabolism, Sheng Yushou Center of Cell Biology and Immunology, School of Life Science and Biotechnology, Shanghai Jiaotong University, Shanghai, USA; 2 Department of Microbiology and Immunology, Weill Cornell Medical College, New York, NY, USA

**Keywords:** IL-12, IL-10, immune signaling

## Abstract

Interleukin-10 and Interleukin-12 are produced primarily by pathogen-activated antigen-presenting cells, particularly macrophages and dendritic cells. IL-10 and IL-12 play very important immunoregulatory roles in host defense and immune homeostasis. Being anti- and pro-inflammatory in nature, respectively, their functions are antagonistically opposing. A comprehensive and in-depth understanding of their immunological properties and signaling mechanisms will help develop better clinical intervention strategies in therapy for a wide range of human disorders. Here, we provide an update on some emerging concepts, controversies, unanswered questions, and opinions regarding the immune signaling of IL-10 and IL-12.

## Interleukin-12 signaling 

Interleukin-12 (IL-12) is the first member of a family of heterodimeric cytokines identified ^1^. It is a pro-inflammatory molecule produced primarily by professional antigen-presenting cells (APCs), including monocytes/macrophages and dendritic cells (DCs) ^2^. IL-12 is composed of p35 (encoded by *Il12a*) and p40 (encoded by *Il12b*) chains, and it principally activates natural killer (NK) cells and induces the differentiation of naïve CD4 ^+^ T lymphocytes to become interferon-gamma (IFN-γ)-producing T helper 1 (Th1) effectors in cell-mediated immune responses to intracellular pathogens ^2^. IFN-γ, in turn, acts on APCs to augment IL-12 secretion in a positive feedback loop ^3, 4^. The p40 chain can also form a dimer with p19 to give rise to IL-23 ^5^, which is required for Th17 differentiation, function, and maintenance ^6^. Similarly, the p35 chain can combine with Epstein-Barr-induced 3 (EBI3) to form IL-35 ^7^, the latest addition to the IL-12 family, in induced regulatory T-cell population (referred to as iTr35 ^8^) and in tolerogenic human DCs ^9^. IL-12 and IL-23 have overlapping as well as distinct immunostimulatory activities ^6^. IL-12 signals through the IL-12 receptor (IL-12R) comprised of the IL-12Rβ1 and IL-12Rβ2 subunits that are expressed on T cells, NK cells, and DCs ^10, 11^. IL-12 stimulates non-receptor Janus kinase 2 (JAK2) and tyrosine kinase 2 (TYK2) activities, leading to the phosphorylation of signal transducers and activators of transcription (STATs) (in particular, STAT4 homodimers) ^12, 13^. IL-35 is an immunosuppressive cytokine that signals through IL-12β2 and gp130, resulting in the heterodimeric formation and activation of STAT1 and STAT4, which in turn bind to the unique promoter regions of *Ebi3* and *Il12a* ^14^.

## Regulation of interleukin-12 production

Both *Il12a* and *Il12b* genes need to be expressed coordinately in the same cells to produce biologically active IL-12 ^15^. Paradoxically, the mRNA of *Il12a* is widely expressed in many cell types, albeit at low levels in some cells, most of which do not even produce IL-12. The *Il12b* mRNA is restricted to cells that can produce biologically active heterodimer ^16^. Synthesis of the p35 chain was proposed to be a rate-limiting step for IL-12 production for its low abundance of transcripts in cells under steady-state conditions ^17^. Over the past 20 years, a large number of molecular analyses have identified numerous transcription factors that bind to the promoter regions of *Il12a* and *Il12b.* The promoters of *Il12a* have been shown to bind transcription factors such as nuclear factor kappa B (NFκB) c-Rel (in DCs) ^18^, c-Maf (as an inhibitor) ^19^, and IFN regulatory factor 1 (IRF-1) ^20^ in activated macrophages. Goriely *et al.* showed that lipopolysaccharide (LPS)- and IFN-γ-induced human *Il12a* gene activation was immediately preceded by a selective and rapid remodeling of a single positioned nucleosome within the -396/-241 region of the promoter containing critical Sp1-binding sites ^21^. The same group also reported that, in human DCs activated through Toll-like receptor 3 (TLR3) and TLR4 but not TLR2, IRF-3 was recruited to an IFN-stimulated response element (ISRE) between -251 and -242 in the *Il12a* gene promoter. Accordingly, DCs from IRF-3-deficient mice were impaired in TLR4-induced *Il12a* mRNA expression and IL-12p70 synthesis ^22^.

Interestingly, a novel nuclear protein called GC-binding protein (GC-BP) was found in macrophages that engulf apoptotic cells via phagocytosis. GC-BP is activated via tyrosine phosphorylation induced by interactions between the phagocyte and the apoptotic cell expressing externalized phosphatidylserine. GC-BP has a direct and selective inhibitory activity on the transcription of the *Il12a* gene and IL-12 production ^23^. It is speculated that this is part of the mechanisms that help suppress autoimmune responses to self-antigens during the clearance of apoptotic cells. This notion is consistent with the converse observation of the induction of IL-10 production during phagocytosis of apoptotic cells ^24^.

Compared with *Il12a*, the *Il12b* promoter has been more extensively studied, and numerous transcriptional factors have been identified as regulators for *Il12b* transcription. When murine macrophages are stimulated with LPS, nucleosome 1 is selectively remodeled so that the transcription factor CCAAT enhancer-binding protein β (C/EBPβ)/LAP could gain access to this region ^25^. However, remodeling of nucleosome 1 alone is not sufficient for *Il12b* transcription and more factors are required for its induced expression. These factors include NFκB ^26, 27^, PU.1 ^28^, IRF-1 ^29^, nuclear factor in activated T cells (NFAT) ^30^, and IFN consensus sequence-binding protein (ICSBP, also called IRF-8) ^31^ in human or murine macrophages or both. Activation protein 1 (AP-1) has been reported to be an activator of *Il12b* transcription in LPS-stimulated macrophages ^32^, whereas in tumor-derived prostaglandin E _2_ (PGE _2_)-treated macrophages, it appears to play the opposite role: inhibiting *Il12b* transcription and promoting tumor progression *in vivo* ^33^. The controversy has not been resolved to date.

Goodridge *et al.* observed that whilst LPS-induced p38 mitogen-activated protein kinase (MAPK) activation is required for the induction of both p40 and p35 subunits, extracellular signal-regulated kinase (ERK) signaling mediates negative feedback regulation of p40, but not p35, production ^34^. Such ERK activation is downstream of calcium influx and targets LPS-induced *Il12b* transcription by suppressing the synthesis of the transcription factor IRF-1. In contrast, the negative regulation of the p35 subunit of IL-12 occurs via a calcium-dependent, but ERK-independent, mechanism, which was thought to involve NFκB signaling.

CpG oligodeoxynucleotides (ODN) activates the TLR9/MyD88/TRAF6 (TNF receptor-associated factor 6) cascade leading to the activation of I kappa B kinase (IKK) -NFκB and JNK, which are critical for the production of pro-inflammatory cytokines. Ma *et al.* reported that the catalytic subunit of DNA-dependent protein kinase (DNA-PKcs) is involved in this activation process ^35^. DNA-PKcs-deficient DCs exhibited a defect in the IL-6 and IL-12p40 expression in response to CpG-ODN in a dose- and time-dependent manner. Loss of DNA-PKcs impaired phosphorylation of IKK, IκBα, NFκB, and JNK in response to CpG-ODN ^35^. TLR2-mediated production of IL-12p40 in monocytes and macrophages triggered by the synthetic ligand Pam3csk4 has been shown to activate the phosphorylation of JNK-1/2. Blocking JNK with a chemical inhibitor resulted in inhibition of Pam3csk4-induced p40 production ^36^. However, the further downstream signaling is not clear.

At the transcriptional level, the differential regulation of *Il12a* and *Il12b* genes is well illustrated in macrophages derived from C/EBPβ-deficient mice. In sharp contrast to the enhanced induction of *Il12b* mRNA, C/EBPβ ^−/−^ primary macrophages derived from both the bone marrow and the peritoneal cavity displayed a totally defective expression of *Il12a* mRNA. This may explain the defective production of bioactive IL-12 and the impaired Th1 responses of C/EBPβ-deficient mice to *Candida albicans*, a pathogen that requires Th1-mediated control ^37^. The enhanced p40 production in C/EBPβ-deficient macrophages is in direct contradiction to an earlier molecular study ^25^. It cautions against directly extrapolating *in vitro* data for its *in vivo* relevance.

An important pathway in robust IL-12 induction is the requirement for “priming” of LPS-activated macrophages and DCs by IFN-γ for the expression of maximal amounts of *Il12a* and *Il12b* mRNAs and for IL-12 production ^4, 20, 38^. The IFN-γ priming is a positive feedback mechanism for more robust IL-12 production in certain immune responses, as the primer IFN-γ is derived principally from NK cells and activated Th1 lymphocytes, cells that are initially activated by APC-derived IL-12 upon pathogen infection. Overall, inadequate investigations have been performed to elucidate this important feedback amplification mechanism in a comprehensive manner.

 Negishi *et al.* reported that MyD88-associated IRF-1 migrates into the nucleus more efficiently than non-MyD88-associated IRF-1. The critical role of MyD88-dependent “IRF-1 licensing” is underscored by the observation that the induction of a specific gene subset downstream of the TLR-MyD88 pathway, such as IFN-β, inducible nitric oxide (NO) synthase, and IL-12p35, is impaired in Irf1-deficient cells ^39^. The study places IRF-1 as an additional member participating in MyD88 signaling and provides a mechanistic explanation for the enhancement of the TLR-dependent IL-12p35 induction program by IFN-γ.

The TLR-NFκB-dependent pathway inducing IL-12 and the IFN-dependent pathway inducing type I IFN (α and β) and IFN-regulated genes have also been shown to cooperate for the robust production of IL-12 in DCs. Gautier *et al.* reported that R-848/Resiquimod (TLR7 ligand in the mouse and TLR7/8 ligand in human) synergized with poly (I:C) (TLR3 ligand) or LPS (TLR4 ligand) in inducing high levels of bioactive IL-12p70 secretion and IFN-β mRNA accumulation by mouse bone marrow-derived DCs (BMDCs). Strikingly, IL-12p70, but not IL-12p40, secretion was strongly reduced in BMDCs from STAT1 ^−/−^ and IFNAR ^−/−^ mice. STAT1 tyrosine phosphorylation, IL-12p35, and IFN-γ mRNA accumulations were strongly inhibited in IFNAR ^−/−^ BMDCs activated with the TLR ligand combinations. Similar observations were made by using neutralizing anti-IFNAR2 antibodies in human TLR8-expressing peripheral blood monocyte-derived DCs ^40^. This study suggests that TLR engagement on DC induces endogenous IFNs that cooperate with the NFκB-inducing machinery for optimal IL-12p70 secretion.

Signaling events from distinct classes of pathogen recognition receptors (PRRs) affect each other in modulating innate and adaptive immunity through modulating IL-12 production. Activation of cytosolic RIG-I-like receptors (RLRs) results in the selective suppression of TLR-induced transcription of the *Il12b* gene through the binding of RLR-activated transcription factor IRF-3 to the *Il12b* promoter, where it competitively edges out IRF-5, a transcriptional activator of *Il12b* that binds to the same sequence motif, the ISRE. IRF-5 binding in this region is usually accompanied with chromatin remodeling of both regulatory regions and the formation of a productive transcriptional complex containing other transcription factors ^41^. Consequently, the activation of RLRs in mice attenuated TLR-induced Th1 and Th17 responses against viral infection of mice ^42^. Similarly, Kim *et al.* identified a crosstalk between TLR4- and nucleotide-binding oligomerization domain 2 (NOD2)-mediated activities in the regulation of intestinal mucosal defense and tissue homeostasis via NOD2 signaling selectively interfering with TLR-induced *Il12a* gene expression and IL-12 production via the transcriptional regulator C/EBPα ^43^.

**Figure 1.**
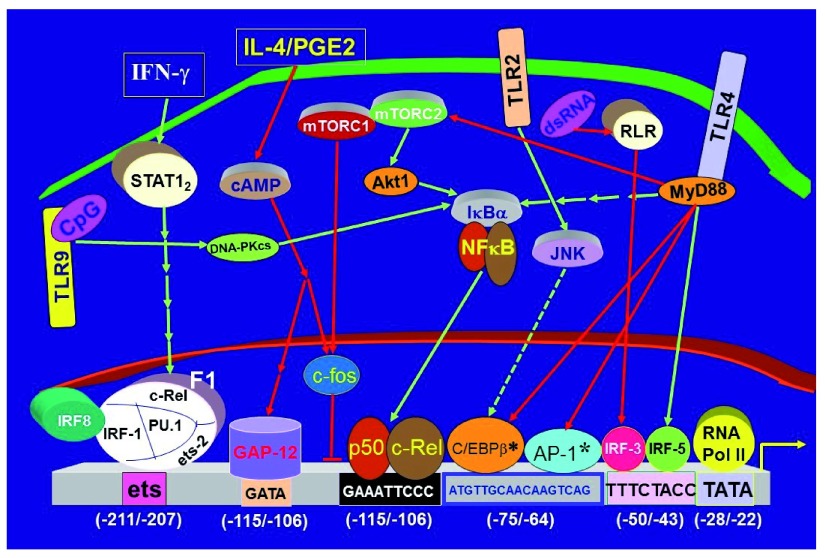
Transcriptional regulation of IL-12p40 (IL12b) in antigen-presenting cells. The data are drawn primarily from macrophage studies. In dendritic cells, c-Rel is not required for IL12B transcription. F1 denotes a large molecular complex containing multiple transcription factors binding to the human IL12b promoter ^47^. Green-arrowed lines indicate a stimulatory role for IL12b transcription, whereas red-arrowed lines denote the reverse. Continuous short arrows denote multiple steps involved that are not specified in details. Dashed lines indicate undetermined signaling pathway. The promoter coordinates are with respect to the transcription start site, designated +1, of the human IL12b gene. GAP-12 is a putative transcriptional repressor of unidentified nature that is induced by IL-4 or PGE2 treatment of human monocytes ^28^. The asterisks denotes controversial transcriptional factors that are defined as repressors by mouse knockout studies but as activators in some *in vitro* studies (see text for details). Akt, Ak strain transforming; AP-1; activating protein 1; cAMP, cyclic adenosine monophosphate; C/EBP, CCAAT enhancer-binding protein; CpG, cytosine-phosphate-guanine; ds, double-stranded; Ets2, E26 2; GAP-12, GATA sequence in the IL-12 promoter; IRF, interferon regulatory factor; JNK, c-Jun N-terminal kinase; MyD88, myeloid differentiation primary response gene 88; mTOR, mammalian target of rapamycin; PGE2, prostaglandin E2; PK, protein kinase; Pol, polymerase; PU.1, purine.1; RLR, retinoic acid-inducible gene-I-like receptor; STAT, signal transduction and transcription; TLR, Toll-like receptor.

Emerging evidence has demonstrated that mammalian target of rapamycin (mTOR) is an important regulator of immunity by modulating the differentiation, activation, and function of lymphocytes and APCs ^44^. In exploring the long-held “puzzle” of low levels of IL-12 induced through TLR4 signaling in macrophages and DCs, which implied the existence of stringent regulatory mechanisms, He *et al.* identified the critical regulatory roles of three protein kinases, mTOR, phosphoinositide-3 kinase (PI3K), and ERK, in TLR-induced Th1 responses by reciprocally controlling IL-12 and IL-10 production in innate immune cells of murine origin ^45^. Moreover, it was revealed that c-fos was a key molecule that mediated the kinase-regulated IL-12 and IL-10 expression in TLR4 signaling by regulating c-fos expression and NFκB binding to the promoters of IL-12 and IL-10 in a differential manner ^45^. These findings confirmed the role of c-fos in this capacity reported in an earlier study by Mitsuhashi *et al.* ^33^ and were corroborated by a similar study in human DCs with an additional delineation of the opposing activities of the two components of the mTOR complex, mTORC1 and mTORC2, in this signaling pathway ^46^. Thus, by controlling the balance between IL-12 and IL-10, mTOR can specifically regulate the TLR-induced T-cell response *in vivo*. Indeed, blockade of mTOR by rapamycin efficiently boosted TLR-induced antigen-specific T- and B-cell responses to hepatitis B virus and hepatitis C virus vaccines ^45^. This study links a ubiquitously present and fundamentally important pathway of cellular survival, proliferation, and function to the production of a highly restricted specialist molecule in the immune system. Notably absent from the study is the answer to an obvious question: is the induction of IL-10 via mTOR signaling responsible for the inhibition of IL-12 production? Figure 1 summarizes our current understanding of the transcriptional mechanisms regulating the IL-12p40 promoter ^47^.

## Interleukin-10 signaling

IL-10 was first discovered by complementary DNA clone-based screening for secreted factors by established Th2 cells that regulate cytokine production by activated Th1 cells ^48, 49^. IL-10 is a major immunosuppressive cytokine. It is a critical component in the maintenance of the fine balance between swift and potent immune responses against invading pathogens and the control of detrimental pathological injury. Almost all cells of the innate and adaptive arms of the immune system can produce IL-10, including DCs, macrophages, mast cells, NK cells, eosinophils, neutrophils, B cells, CD8 ^+^ T cells, CD4 ^+^ Th1, Th2, and Th17 cells ^50– 58^, and regulatory T (Treg) cells ^53, 57, 59^. The major role of IL-10 is to limit the extent of the activation of both the innate and the adaptive immune cells to maintain a homeostatic state. This role of IL-10 is vitally important in protecting the host from infection-associated immunopathology, autoimmunity, and allergy, such as sepsis, arthritis, insulitis, inflammatory bowel disease (IBD), and so on. In addition to these activities, IL-10 regulates growth or differentiation (or both) of B cells, NK cells, cytotoxic and helper T cells, mast cells, granulocytes, dendritic cells, keratinocytes, and endothelial cells ^51^.

The IL-10 receptor is composed of at least two subunits that are members of the IFN receptor (IFNR) family, the ligand-binding subunit (IL-10Rα and IL-10R1) ^60, 61^, and the accessory subunit for signaling, IL-10R2 (IL-10Rβ) ^62, 63^. IL-10, produced from various cellular sources upon exposure to pathogens and inflammatory insults, binds to its receptor on target cells. Activation of the IL-10 receptor complex induces a tetramer consisting of two IL-10R1 and two IL-10R2 chains, which bind homodimeric IL-10 to the extracellular domains of IL-10R1 ^64^. Upon the receptor-ligand engagement, phosphorylation of the receptor-associated protein tyrosine kinase JAK1 is recruited to the intracellular domain by the IL-10R1 chain, while non-receptor TYK2 is recruited to the receptor complex by IL-10R2 ^62^. These kinases serve as a temporary docking site for inactive cytosolic STAT1 or STAT3 or both ^62^, which are recruited by JAK1 and TYK2 to the site upon phosphorylation of the IL-10R1 chain at two tyrosine residues ^64^. The STATs bind to the IL-10R1 chain via the *Src* homology 2 (SH2) domain and are tyrosine-phosphorylated by the receptor-associated JAKs. Activation of STAT3 leads to its homodimerization, similarly to STAT1 ^65, 66^. Translocation of activated STATs to the nucleus renders high-affinity binding to the promoter regions of IL-10-responsive genes. Successful engagement of the IL-10 receptor complex subsequently activates distinct JAK-STAT pathways and downstream signaling events that converge through various mechanisms to influence nuclear transcriptional events such as those mediated by NFκB ^67^, resulting in the initiation of extensive anti-inflammatory and homeostatic programs.

It is important to note that the cellular source of IL-10 production is critical to its immunological activities in a cell-specific manner. Mice with a specific deletion in T cells generated by Cre/loxP-mediated targeting showed heightened contact hypersensitivity reactions and succumbed to severe immunopathology upon infection with *Toxoplasma gondii*. Splenocytes from these mice secreted increased amounts of pro-inflammatory cytokines after activation *in vitro* compared with wild-type (WT) control splenocytes. However, in contrast to complete IL-10 deficiency, sensitivity to endotoxic shock and skin irritant responses of the skin in the T-specific IL-10-deficent mice were not greater than those of the WT controls ^68^. A critical role of B cell-derived IL-10 has been demonstrated in the mouse model of experimental autoimmune encephalomyelitis (EAE). Mice with a disruption in the Ig μ heavy chain (μMT), which results in a lack of B cells, develop a non-remitting form of EAE. Transfer of WT B cells restored remission, whereas B lymphocytes from IL-10-deficient mice were unable to suppress the disease progression ^52^. Together, these studies highlight the distinctiveness of IL-10 derived from different cellular origins that determines its unique range of activities.

## Regulation of interleukin-10 production

IL-10 production by macrophages and DCs through pathogen-associated molecular patterns (PAMPs) has been most widely studied. Macrophages produce IL-10 as a consequence of the recognition of PAMPs by its PRRs. Several classes of PRRs are expressed by macrophages, including TLRs, C-type lectin receptors, RIG-1 (retinoic acid-inducible gene 1) receptors, and NOD-like receptors ^69, 70^. The PAMPs bind to the TLRs with its TLR-interacting (TIR) domain, initiating signaling into macrophages with the help of intracellular adaptors that lead to the activation of multiple members of the MAPKs and subsequently transcription factors Sp1 ^71^, C/EBPβ and δ ^72^, c-Maf ^73^, NFκB ^74^, and phosphorylated cyclic AMP element-binding protein (CREB) ^75^. TLRs can also act in synergy with other agonists such as IL-4 ^73^ and PGE _2_^76, 77^ to augment IL-10 production. TLR3 or TLR4 activation results in the production of IFNβ, which sets up a feedback loop to sustain IL-10 mRNA induction ^78^.

B cells express a number of TLRs. Agonists that act via TLR2, TLR4, or TLR9 have all been shown to promote IL-10 production ^79– 82^. TLR9 activation in B cells stimulates activation of Bruton’s tyrosine kinase (Btk), and B cells from Btk knockout mice fail to secrete IL-10 following TLR9 stimulation. However, the molecular mechanism downstream of Btk is not clear. The role of Btk is not restricted to B cells, as Btk-deficient macrophages also secrete less IL-10 than WT cells ^83^.

CD4 ^+^ T cells have been identified as an important source of IL-10 *in vivo* ^84^. Various transcription factors have been reported to induce IL-10 in T cells, including SP1, c-Jun, c-Maf, SMAD4, GATA3, and STATs ^84^. However, the molecular signaling pathways that regulate IL-10 induction have not been fully delineated. The studies in this area have been complicated by the existence of multiple Th cell subsets, many of which can produce IL-10, including Th1, Th2, Th17, and Treg cells, albeit with different capacities. These observations have prompted the hypothesis that the IL-10 locus becomes differentially modified during Th cell polarization, which then invokes subtly different molecular mechanisms that drive IL-10 transcription in a quantitatively variable manner in the various T-cell subtypes ^85^.

In contrast to the host response to infectious agents, clearance of apoptotic cells of a self-nature by phagocytes results predominantly in anti-inflammatory reactions characterized by the production of immunoregulatory cytokines IL-10, PGE _2_, and transforming growth factor beta (TGFβ) ^86^, which are critical to ensuring cellular homeostasis and suppression of autoimmunity as an evolutionarily well-preserved mechanism. Chung *et al.* reported that the production of IL-10 in response to apoptotic cells is dependent on CD36, p38 MAPK, and the transcription factor TALE homeoprotein Pre-B-cell leukemia homeobox 1 (Pbx1) ^24^. The study establishes a novel role of a developmentally critical factor in the regulation of homeostasis in the immune system and opens up a new area for future exploration at the intersection between cellular homeostasis and immune responses to exogenous pathogens as well as to endogenous danger signals.

## Regulation of interleukin-12 production by interleukin-10

The potency of IL-12 in host defense makes it a target for stringent regulation. Indeed, the temporal, spatial, and quantitative expression of IL-12 during an immune response in a microenvironment contributes critically to the determination of the type, extent, and ultimate resolution of the reaction. Breaching of the delicate control and balance frequently leads to immunologic disorders and pathogenesis. One of the most important and well-studied negative regulators of TLR-induced IL-12 production is IL-10 ^87^. IL-10 suppression of both *IL12a* and *Il12b* genes is seen primarily at the transcriptional level, and the inductions of the two genes have different requirements for *de novo* protein synthesis ^88^. How IL-10 suppresses *Il12a* transcription is unknown at present. IL-10 targets an enhancer 10 kb upstream of the *Il12b* transcriptional start site that is bound by nuclear factor, interleukin 3-regulated (NFIL3), a B-ZIP transcription factor. Myeloid cells lacking NFIL3 produce excessive IL-12p40 and increased IL-12p70 ^89^. Thus, the STAT3-dependent expression of NFIL3 is a key component of a negative feedback pathway in myeloid cells that suppresses pro-inflammatory responses.

Kobayashi *et al.* observed that acetylated histone H4 transiently associated with the *Il12b* promoter in WT bone marrow-derived macrophages (BMDMs), whereas association of these factors was prolonged in Il10 ^−/−^ BMDMs. Experiments using histone deacetylase (HDAC) inhibitors and HDAC3 short hairpin RNA indicate that HDAC3 is involved in histone deacetylation of the *Il12b* promoter by IL-10. These results suggest that histone deacetylation on the *Il12b* promoter by HDAC3 mediates the homeostatic effect of IL-10 in macrophages ^90^. More details clearly need to be worked out to understand the important homeostatic regulation of IL-12 production by IL-10. In this context, the IL-4-inducing transcription factor c-Maf is an interesting molecule that can directly and conversely regulate IL-12 and IL-10 gene expression in activated macrophages ^19, 91^. Conversely, IRF-5 is a driver of the “M1” polarization of macrophages promoting Th1 and Th17 activities with activated transcription of inflammatory genes, including *Il12a*, *Il12b*, and *Il23a*, and repressed *Il10* transcription ^92^.

## Interleukin-12 in adoptive cell therapy for cancer

IL-12 is able to activate all major cytotoxic killer and helper cell types of the immune apparatus (NK, NKT, CD4 ^+^, and CD8 ^+^ T cells) that are crucially important for immunosurveillance of and resistance to cancer development and progression ^93^. The extraordinary anti-tumor efficacy of IL-12 has been demonstrated in animal models of cancer of diverse types ^94– 105^, and its use in various forms is now involved in a large number of human cancer clinical trials ^106^. Adoptive cell therapy of malignant diseases takes advantage of the cellular immune system to recognize specific tumor-associated antigens and destroy cancer cells. This is remarkably demonstrated by redirecting T cells with a chimeric antigen receptor (CAR) toward CD19, inducing complete remission of leukemia in more than two thirds of patients in early-phase trials ^107^. After initial tumor reduction by CAR T cells, antigen-negative cancer cells not recognized by CAR may give rise to tumor relapse. Fortunately, the “quagmire” may be overcome by CAR-mediated activation of T cells in the tumor, releasing inducible IL-12, which augments T-cell activation and attracts and activates innate immune cells to eliminate antigen-negative cancer cells in the targeted lesion. Chmielewski *et al.* demonstrated the feasibility of this strategy by redirecting T cells with a carcinoembryonic antigen (CEA)-targeting CAR and engineering with the inducible recombinant IL-12 expression cassette under the control of the NFAT/IL-2 minimal promoter ^108^. In this context, IL-12 release was triggered by CAR signaling upon tumor antigen recognition and no IL-12 was detected *in vitro* without CAR signaling. The production capacity of such modified CAR T cells was sufficient to reach therapeutic levels without the need of repetitive drug application ^109^. The therapeutic advantage is indicated by the fact that a dose of 10 ^5^ IL-12 modified tumor-specific CAR T cells was more effective against established tumors than 10 ^6^ T cells without IL-12 in a pre-clinical model ^110^.

To date, despite the enhanced anti-tumor efficacy of IL-12-secreting CAR T cells in this model, the mechanisms associated with this enhanced tumor eradication remain unclear. Previous work showed that IL-12 reversed Treg cell-mediated suppression of CD4 ^+^ Foxp3 ^−^ T-cell proliferation ^111^. IL-12 was shown to induce IFN-γ production by Treg cells *in vitro* and *in vivo* ^112, 113^. However, IFN-γ expression did not decrease the ability of Treg cells to suppress T-cell proliferation ^114^. Rather, IL-12 treatment decreased Treg cell frequency and Foxp3 levels in Treg cells. Furthermore, IL-12 increased IL-2R expression on effector CD4 ^+^ and CD8 ^+^ T cells, diminished its expression on Treg cells, and decreased IL-2 production by CD4 ^+^ and CD8 ^+^ T effectors. Together, these IL-12-mediated changes favored the outgrowth of non-Treg cells ^114^. Kerkar *et al.* demonstrated that engineering tumor-specific CD8 ^+^ T cells to secrete IL-12 improved their therapeutic efficacy in the B16 mouse model of established melanoma ^115^. Surprisingly, direct binding of IL-12 to receptors on lymphocytes or NK cells was not required. Instead, IL-12 sensitized bone marrow-derived tumor stromal cells, including CD11b ^+^F4/80 ^hi^ macrophages, CD11b ^+^MHCII ^hi^CD11c ^hi^ DCs, and CD11b ^+^Gr-1 ^hi^ MDSCs, causing them to enhance the effects of adoptively transferred CD8 ^+^ T cells. This reprogramming of myeloid-derived cells occurred partly through IFN-γ. MHC I expression on host cells was essential for IL-12-mediated anti-tumor enhancements ^115^. These studies point to the potential immunological mechanisms of the T cell-secreted IL-12 in tumor models.

Based on prior pre-clinical studies demonstrating that IL-12-secreting CAR T cells are protected from inhibition by endogenous Treg cells (unpublished results), it is conceivable that IL-12-producing CAR T cells may be refractory to Treg cell-mediated inhibition and that previously requisite CAR-mediated T-cell “co-stimulation” (through CD28 or CD40L) may be overcome by CAR T cell-derived IL-12 secretion. In other words, CAR T cell-derived IL-12 may render the effectors independent of the “second signal” requirement “engraved” in classic T-cell activation paradigms. Furthermore, it is possible that IL-12 secretion within the tumor microenvironment can reverse the anergic state of endogenous tumor-infiltrating lymphocytes (TILs) and blunt the immune suppression by myeloid-derived suppressor cells (MDSCs) as well as modulation of the tumor-associated macrophages (TAMs) from a suppressive M2 phenotype to a pro-inflammatory M1 phenotype ^116– 119^.

## Future perspectives

IL-10 is a pleiotropic cytokine with a strong role in limiting the scope and extent of immune activation. Loss of IL-10 function has deleterious effects. Therefore, IL-10 could be a potential therapeutic agent for many inflammatory or autoimmune disorders. However, systemic IL-10 administration has proven to be of limited value ^120^ and this indicates that IL-10 production would need to be carefully targeted to be efficacious therapeutically. This is evidenced by adoptive transfers of specific types of IL-10-producing immune cells in some autoimmune disease models that result in protection against the development of inflammatory pathologies ^121– 127^. Thus, a far more comprehensive and precise understanding of which IL-10-producing cells are important *in vivo*, and what the critical target cells of this IL-10 are would be instrumental in the future development of the therapeutic potentials of IL-10. The increased use of conditional gene targeting in mice will help in these future studies ^85^.

In the intestinal mucosa, IL-10 is a well-established regulator of tissue inflammation and homeostasis. Mutations in the NOD2 gene are strongly associated with Crohn’s disease, a form of IBD believed to be driven by uncontrolled Th1 and Th17 responses ^128^. There has been long a debate on the nature of the IBD-associated NOD2 mutations: “loss of function” or “gain of function”? Noguchi *et al.* showed that a common disease-related NOD2 mutation, 3020insC, displayed a “gain of function” property in that it suppressed *IL10* transcription by blocking the phosphorylation of the nuclear ribonucleoprotein hnRNP-A1 (heterogeneous nuclear ribonucleoprotein A1) via the p38 MAPK ^129^. This effect of 3020insC appears to be unique on the human IL-10 gene but not on its murine counterpart. The study challenges the present paradigms about the influence of the 3020insC mutation on Crohn’s disease, cautioning against deriving conclusions about the human disease on the basis of data from NOD2 knockout mice. It may provide a novel way of thinking about efforts to identify therapeutic targets for the treatment of Crohn’s disease and other Th1/Th17-mediated autoimmune diseases associated with the 3020insC mutation.

Although a tremendous amount of knowledge has been gained about the signaling and function of IL-12 in immune cells since its discovery in 1989, many important questions remain. It is widely believed that the majority of the immunological activities of IL-12 are mediated through IFN-γ produced by activated NK and Th1 cells that have been exposed to APC-derived IL-12. However, considerable levels of IFN-γ-independent activities of IL-12 have been reported in many infectious disease and cancer models ^130– 136^. The cellular and molecular basis of the non-canonical activities of IL-12 await further elucidation. In immunotherapy of cancers, it has been long noted that the repeated administration of recombinant IL-12 could contribute to increased immunosuppressive properties of the tumor by the induction of IL-10 ^137– 139^. Although the underlying molecular mechanism for the negative feedback is lacking, the finding that IL-12 is capable of potently inducing its own inhibitor reiterates the concept that the immune system is inherently equipped with an intrinsic negative feedback device that limits ongoing T-cell activation. This also indicates that the kinetics of T-cell responses may be regulated by the ratio of IL-12 and IL-10 levels, which may gradually decline during the immune response.

Endotoxin tolerance, the transient, secondary downregulation of a subset of endotoxin-driven responses after exposure to bacterial products, is thought to be an adaptive response providing protection from pathological hyperactivation of the innate immune system during bacterial infection. IL-12 production is subjected to such a control mechanism. Wysocka *et al.* examined the development of IL-12 suppression during endotoxin tolerance in mice. The basis for decreased IL-12 production *in vivo* is clearly multifactorial, involving both loss of CD11c ^high^ DCs as well as alterations in the responsiveness of macrophages and remaining splenic DCs. There is no demonstrable mechanistic role for B or T lymphocytes, the soluble mediators IL-10, TNF-α, IFN-α/β, nitric oxide, or the NFκB family members p50, p52, or RelB ^140^. To date, the tolerance mechanism that inhibits IL-12 production by APCs remains elusive. The need for the understanding is underscored by the frequent occurrence of “immunological paralysis” subsequent to septic shock in patients. In the broad context, Foster *et al.* have provided some major insights into this phenomenon by proposing a model for the gene-specific regulation of class “T” (for tolerant) and “NT” (for non-tolerant) genes mainly through preferential transcription factor recruitment, histone acetylation, H3K4 trimethylation, and chromatin remodeling in tolerant versus non-tolerant macrophages ^141^.

The acute-phase proteins, C-reactive protein and serum amyloid A (SAA), are biomarkers of infection and inflammation. He *et al.* reported a novel property of SAA in the differential induction of IL-12 and IL-23 in human peripheral blood monocytes ^142^. SAA-induced IL-12p40 production was accompanied by a sustained expression of IL-23p19, but not IL-12p35, resulting in preferential secretion of IL-23, but not IL-12. The study identified SAA as a novel endogenous ligand that potentially activates the IL-23/IL-17 pathway, representing a novel mechanism for regulation of inflammation and immunity by an acute-phase protein. The differential production of IL-12 versus IL-23 was also observed in myeloid DCs (mDCs) and plasmacytoid DCs (pDCs) stimulated via TLR ligands. Only mDCs but not pDCs secreted IL-23. Although pDCs produced both mRNA and protein of the p40 subunit, the lack of bioactive heterodimeric IL-23 protein release was due to the absence of translation of the p19 mRNA into protein ^143^. These findings support the hypothesis of a coordinated adaptive immune response based on a finely tuned contribution of these cytokines by different DC subsets. How these endogenous and exogenous ligands induce IL-12 and IL-23 differentially at the molecular level bears both great scientific interests and practical implications.

The immunological activities of IL-12 are further complicated by the existence of IL-12p40 homodimer, IL-12p80, which acts as an IL-12 antagonist by binding to the IL-12R but which does not mediate a biological response ^144, 145^. Secretion of IL-12 is associated with excess production of IL-12p80 ^16^. For example, in contrast to the dogma about the restrictive nature of IL-12-producing cell types, meaningful amounts of IL-12p40 monomer and IL-12p80 have been observed in human breast cancer cells ^146^, which could potentially thwart the IL-12-induced anti-tumor responses *in vivo*. Approximately 20% to 40% of the p40 in the serum of normal and endotoxin-treated mice is in the form of IL-12p80 ^147^. In IL-12-dependent shock models, exogenous IL-12p80 inhibits IL-12-induced cell-mediated immune response and protects mice from sepsis-associated death ^148^. However, IL-12p80 has also been reported to stimulate, rather than inhibit, the differentiation of CD8 ^+^ Tc1 (type I cytotoxic T) cells *in vitro*, contrary to its suppressive activity on Th1 function ^149^. The divergent functions of the various forms of p40 highlight our lack of understanding of its true range of biological activities.

Recent pre-clinical studies demonstrated that treatment with CD19-specific, CAR T cells that secrete IL-12 is able to safely eradicate established disease without the sophisticated and laborious prior conditioning of subjects ^150^. Moreover, in severe combined immunodeficient (SCID)-Beige mice with human ovarian cancer xenografts, IL-12-secreting CAR T cells exhibited enhanced anti-tumor efficacy as determined by an increased survival rate, prolonged persistence of T cells, a higher level of systemic IFN-γ, and modulated tumor microenvironment ^151^. How the locally released IL-12 contributes to the highly favorable clinical efficacy and immunological modifications to numerous cell types in the tumor environment is an urgent and challenging task for the benefit of further improving this revolutionary therapeutic strategy for cancers of diverse types and progression states.

In summary, the complexity of the heterodimeric nature of both the cytokines and their receptors in the IL-12 family (also including IL-27) associated with the activation of different combinations of tyrosine kinases and STATs underlies the overlapping as well as distinct immunological consequences of the regulation and signaling in this cytokine group. Greater efforts are called for to better decipher the intricacies. In the meantime, more caution is needed in interpreting data derived from studies of individual cytokine or receptor chains.

## References

[1] Kobayashi M Fitz L Ryan M: Identification and purification of natural killer cell stimulatory factor (NKSF), a cytokine with multiple biologic effects on human lymphocytes. *J Exp Med.* 1989;170(3):827–45. 2504877 10.1084/jem.170.3.827 PMC2189443

[2] Trinchieri G: Interleukin-12: a cytokine produced by antigen-presenting cells with immunoregulatory functions in the generation of T-helper cells type 1 and cytotoxic lymphocytes. *Blood.* 1994;84(12):4008–27. 7994020

[3] Grohmann U Belladonna ML Vacca C: Positive regulatory role of IL-12 in macrophages and modulation by IFN-gamma. *J Immunol.* 2001;167(1):221–7. 10.4049/jimmunol.167.1.221 11418652

[4] Ma X Chow JM Gri G: The interleukin 12 p40 gene promoter is primed by interferon gamma in monocytic cells. *J Exp Med.* 1996;183(1):147–57. 10.1084/jem.183.1.147 8551218 PMC2192398

[5] Oppmann B Lesley R Blom B: Novel p19 protein engages IL-12p40 to form a cytokine, IL-23, with biological activities similar as well as distinct from IL-12. *Immunity.* 2000;13(5):715–25. 10.1016/S1074-7613(00)00070-4 11114383

[6] Teng MW Bowman EP McElwee JJ: IL-12 and IL-23 cytokines: from discovery to targeted therapies for immune-mediated inflammatory diseases. *Nat Med.* 2015;21(7):719–29. 10.1038/nm.3895 26121196

[7] Collison LW Workman CJ Kuo TT: The inhibitory cytokine IL-35 contributes to regulatory T-cell function. *Nature.* 2007;450(7169):566–9. 10.1038/nature06306 18033300

[8] Sawant DV Hamilton K Vignali DA: Interleukin-35: Expanding Its Job Profile. *J Interferon Cytokine Res.* 2015;35(7):499–512. 10.1089/jir.2015.0015 25919641 PMC4507123

[9] Dixon KO van der Kooij SW Vignali DA: Human tolerogenic dendritic cells produce IL-35 in the absence of other IL-12 family members. *Eur J Immunol.* 2015;45(6):1736–47. 10.1002/eji.201445217 25820702 PMC4617619

[10] Grohmann U Belladonna ML Bianchi R: IL-12 acts directly on DC to promote nuclear localization of NF-kappaB and primes DC for IL-12 production. *Immunity.* 1998;9(3):315–23. 10.1016/S1074-7613(00)80614-7 9768751

[11] Desai BB Quinn PM Wolitzky AG: IL-12 receptor. II. Distribution and regulation of receptor expression. *J Immunol.* 1992;148(10):3125–32. 1578139

[12] Watford WT Hissong BD Bream JH: Signaling by IL-12 and IL-23 and the immunoregulatory roles of STAT4. *Immunol Rev.* 2004;202(1):139–56. 10.1111/j.0105-2896.2004.00211.x 15546391

[13] Trinchieri G: Interleukin-12 and the regulation of innate resistance and adaptive immunity. *Nat Rev Immunol.* 2003;3(2):133–46. 10.1038/nri1001 12563297

[14] Collison LW Delgoffe GM Guy CS: The composition and signaling of the IL-35 receptor are unconventional. *Nat Immunol.* 2012;13(3):290–9. 10.1038/ni.2227 22306691 PMC3529151

[15] Wolf SF Temple PA Kobayashi M: Cloning of cDNA for natural killer cell stimulatory factor, a heterodimeric cytokine with multiple biologic effects on T and natural killer cells. *J Immunol.* 1991;146(9):3074–81. 1673147

[16] D'Andrea A Rengaraju M Valiante NM: Production of natural killer cell stimulatory factor (interleukin 12) by peripheral blood mononuclear cells. *J Exp Med.* 1992;176(5):1387–98. 1357073 10.1084/jem.176.5.1387 PMC2119437

[17] Snijders A Hilkens CM van der Pouw Kraan TC: Regulation of bioactive IL-12 production in lipopolysaccharide-stimulated human monocytes is determined by the expression of the p35 subunit. *J Immunol.* 1996;156(3):1207–12. 8557999

[18] Grumont R Hochrein H O'Keeffe M: c-Rel regulates interleukin 12 p70 expression in CD8 ^+^ dendritic cells by specifically inducing *p35* gene transcription. *J Exp Med.* 2001;194(8):1021–32. 10.1084/jem.194.8.1021 11602633 PMC2193517

[19] Homma Y Cao S Shi X: The Th2 transcription factor c-Maf inhibits IL-12p35 gene expression in activated macrophages by targeting NF-kappaB nuclear translocation. *J Interferon Cytokine Res.* 2007;27(9):799–808. 10.1089/jir.2007.0006 17892401

[20] Liu J Cao S Herman LM: Differential regulation of interleukin (IL)-12 p35 and p40 gene expression and interferon (IFN)-gamma-primed IL-12 production by IFN regulatory factor 1. *J Exp Med.* 2003;198(8):1265–76. 10.1084/jem.20030026 14568984 PMC2194226

[21] Goriely S Demonté D Nizet S: Human *IL-12(p35)* gene activation involves selective remodeling of a single nucleosome within a region of the promoter containing critical Sp1-binding sites. *Blood.* 2003;101(12):4894–902. 10.1182/blood-2002-09-2851 12576336

[22] Goriely S Molle C Nguyen M: Interferon regulatory factor 3 is involved in Toll-like receptor 4 (TLR4)- and TLR3-induced IL-12p35 gene activation. *Blood.* 2006;107(3):1078–84. 10.1182/blood-2005-06-2416 16219795

[23] Kim S Elkon KB Ma X: Transcriptional suppression of interleukin-12 gene expression following phagocytosis of apoptotic cells. *Immunity.* 2004;21(5):643–53. 10.1016/j.immuni.2004.09.009 15539151

[24] Chung EY Liu J Homma Y: Interleukin-10 expression in macrophages during phagocytosis of apoptotic cells is mediated by homeodomain proteins Pbx1 and Prep-1. *Immunity.* 2007;27(6):952–64. 10.1016/j.immuni.2007.11.014 18093541 PMC2194654

[25] Plevy SE Gemberling JH Hsu S: Multiple control elements mediate activation of the murine and human interleukin 12 p40 promoters: evidence of functional synergy between C/EBP and Rel proteins. *Mol Cell Biol.* 1997;17(8):4572–88. 10.1128/MCB.17.8.4572 9234715 PMC232311

[26] Murphy TL Cleveland MG Kulesza P: Regulation of interleukin 12 p40 expression through an NF-kappa B half-site. *Mol Cell Biol.* 1995;15(10):5258–67. 10.1128/MCB.15.10.5258 7565674 PMC230773

[27] Sanjabi S Hoffmann A Liou HC: Selective requirement for c-Rel during IL-12 P40 gene induction in macrophages. *Proc Natl Acad Sci U S A.* 2000;97(23):12705–10. 10.1073/pnas.230436397 11058167 PMC18828

[28] Becker C Wirtz S Ma X: Regulation of IL-12 p40 promoter activity in primary human monocytes: roles of NF-kappaB, CCAAT/enhancer-binding protein beta, and PU.1 and identification of a novel repressor element (GA-12) that responds to IL-4 and prostaglandin E _2_. *J Immunol.* 2001;167(5):2608–18. 10.4049/jimmunol.167.5.2608 11509602

[29] Maruyama S Sumita K Shen H: Identification of IFN regulatory factor-1 binding site in IL-12 p40 gene promoter. *J Immunol.* 2003;170(2):997–1001. 10.4049/jimmunol.170.2.997 12517966

[30] Zhu C Rao K Xiong H: Activation of the murine interleukin-12 p40 promoter by functional interactions between NFAT and ICSBP. *J Biol Chem.* 2003;278(41):39372–82. 10.1074/jbc.M306441200 12876285

[31] Wang IM Contursi C Masumi A: An IFN-gamma-inducible transcription factor, IFN consensus sequence binding protein (ICSBP), stimulates IL-12 p40 expression in macrophages. *J Immunol.* 2000;165(1):271–9. 10.4049/jimmunol.165.1.271 10861061

[32] Zhu C Gagnidze K Gemberling JH: Characterization of an activation protein-1-binding site in the murine interleukin-12 p40 promoter. Demonstration of novel functional elements by a reductionist approach. *J Biol Chem.* 2001;276(21):18519–28. 10.1074/jbc.M100440200 11279072

[33] Mitsuhashi M Liu J Cao S: Regulation of interleukin-12 gene expression and its anti-tumor activities by prostaglandin E _2_ derived from mammary carcinomas. *J Leukoc Biol.* 2004;76(2):322–32. 10.1189/jlb.1203641 15123779 PMC2965202

[34] Goodridge HS Harnett W Liew FY: Differential regulation of interleukin-12 p40 and p35 induction via Erk mitogen-activated protein kinase-dependent and -independent mechanisms and the implications for bioactive IL-12 and IL-23 responses. *Immunology.* 2003;109(3):415–25. 10.1046/j.1365-2567.2003.01689.x 12807488 PMC1782981

[35] Ma C Muranyi M Chu CH: Involvement of DNA-PKcs in the IL-6 and IL-12 response to CpG-ODN is mediated by its interaction with TRAF6 in dendritic cells. *PLoS One.* 2013;8(3):e58072. 10.1371/journal.pone.0058072 23533581 PMC3606245

[36] Wang S Chen Z Hu C: Hepatitis B virus surface antigen selectively inhibits TLR2 ligand-induced IL-12 production in monocytes/macrophages by interfering with JNK activation. *J Immunol.* 2013;190(10):5142–51. 10.4049/jimmunol.1201625 23585678

[37] Gorgoni B Maritano D Marthyn P: *C/EBP* beta gene inactivation causes both impaired and enhanced gene expression and inverse regulation of IL-12 p40 and p35 mRNAs in macrophages. *J Immunol.* 2002;168(8):4055–62. 10.4049/jimmunol.168.8.4055 11937564

[38] Hayes MP Wang J Norcross MA: Regulation of interleukin-12 expression in human monocytes: selective priming by interferon-gamma of lipopolysaccharide-inducible p35 and p40 genes. *Blood.* 1995;86(2):646–50. 7605994

[39] Negishi H Fujita Y Yanai H: Evidence for licensing of IFN-gamma-induced IFN regulatory factor 1 transcription factor by MyD88 in Toll-like receptor-dependent gene induction program. *Proc Natl Acad Sci U S A.* 2006;103(41):15136–41. 10.1073/pnas.0607181103 17018642 PMC1586247

[40] Gautier G Humbert M Deauvieau F: A type I interferon autocrine-paracrine loop is involved in Toll-like receptor-induced interleukin-12p70 secretion by dendritic cells. *J Exp Med.* 2005;201(9):1435–46. 10.1084/jem.20041964 15851485 PMC2213193

[41] Koshiba R Yanai H Matsuda A: Regulation of cooperative function of the *Il12b* enhancer and promoter by the interferon regulatory factors 3 and 5. *Biochem Biophys Res Commun.* 2013;430(1):95–100. 10.1016/j.bbrc.2012.11.006 23154183

[42] Negishi H Yanai H Nakajima A: Cross-interference of RLR and TLR signaling pathways modulates antibacterial T cell responses. *Nat Immunol.* 2012;13(7):659–66. 10.1038/ni.2307 22610141

[43] Kim H Zhao Q Zheng H: A novel crosstalk between TLR4- and NOD2-mediated signaling in the regulation of intestinal inflammation. *Sci Rep.* 2015;5: 12018. 10.1038/srep12018 26153766 PMC4495563

[44] Powell JD Pollizzi KN Heikamp EB: Regulation of immune responses by mTOR. *Annu Rev Immunol.* 2012;30:39–68. 10.1146/annurev-immunol-020711-075024 22136167 PMC3616892

[45] He L Zang A Du M: mTOR regulates TLR-induced c-fos and Th1 responses to HBV and HCV vaccines. *Virol Sin.* 2015;30(3):174–89. 10.1007/s12250-015-3606-3 26122641 PMC8200861

[46] Wei WC Liu CP Yang WC: Mammalian target of rapamycin complex 2 (mTORC2) regulates LPS-induced expression of IL-12 and IL-23 in human dendritic cells. *J Leukoc Biol.* 2015;97(6):1071–80. 10.1189/jlb.2A0414-206RR 25877925

[47] Ma X Neurath M Gri G: Identification and characterization of a novel Ets-2-related nuclear complex implicated in the activation of the human interleukin-12 p40 gene promoter. *J Biol Chem.* 1997;272(16):10389–95. 10.1074/jbc.272.16.10389 9099678

[48] Moore KW Vieira P Fiorentino DF: Homology of cytokine synthesis inhibitory factor (IL-10) to the Epstein-Barr virus gene BCRFI. *Science.* 1990;248(4960):1230–4. 10.1126/science.2161559 2161559

[49] Fiorentino DF Bond MW Mosmann TR: Two types of mouse T helper cell. IV. Th2 clones secrete a factor that inhibits cytokine production by Th1 clones. *J Exp Med.* 1989;170(6):2081–95. 10.1084/jem.170.6.2081 2531194 PMC2189521

[50] Maloy KJ Powrie F: Regulatory T cells in the control of immune pathology. *Nat Immunol.* 2001;2(9):816–22. 10.1038/ni0901-816 11526392

[51] Moore KW de Waal Malefyt R Coffman RL: Interleukin-10 and the interleukin-10 receptor. *Annu Rev Immunol.* 2001;19:683–765. 10.1146/annurev.immunol.19.1.683 11244051

[52] Fillatreau S Sweenie CH McGeachy MJ: B cells regulate autoimmunity by provision of IL-10. *Nat Immunol.* 2002;3(10):944–50. 10.1038/ni833 12244307

[53] Roncarolo MG Gregori S Battaglia M: Interleukin-10-secreting type 1 regulatory T cells in rodents and humans. *Immunol Rev.* 2006;212:28–50. 10.1111/j.0105-2896.2006.00420.x 16903904

[54] O'Garra A Vieira P: T _H_1 cells control themselves by producing interleukin-10. *Nat Rev Immunol.* 2007;7(6):425–8. 10.1038/nri2097 17525751

[55] Trinchieri G: Interleukin-10 production by effector T cells: Th1 cells show self control. *J Exp Med.* 2007;204(2):239–43. 10.1084/jem.20070104 17296790 PMC2118719

[56] Maynard CL Weaver CT: Diversity in the contribution of interleukin-10 to T-cell-mediated immune regulation. *Immunol Rev.* 2008;226:219–33. 10.1111/j.1600-065X.2008.00711.x 19161427 PMC2630587

[57] Sabatos-Peyton CA Verhagen J Wraith DC: Antigen-specific immunotherapy of autoimmune and allergic diseases. *Curr Opin Immunol.* 2010;22(5):609–15. 10.1016/j.coi.2010.08.006 20850958 PMC2977065

[58] Mauri C Bosma A: Immune regulatory function of B cells. *Annu Rev Immunol.* 2012;30:221–41. 10.1146/annurev-immunol-020711-074934 22224776

[59] O'Garra A Vieira P: Regulatory T cells and mechanisms of immune system control. *Nat Med.* 2004;10(8):801–5. 10.1038/nm0804-801 15286781

[60] Tan JC Indelicato SR Narula SK: Characterization of interleukin-10 receptors on human and mouse cells. *J Biol Chem.* 1993;268(28):21053–9. 8407942

[61] Liu Y Wei SH Ho AS: Expression cloning and characterization of a human IL-10 receptor. *J Immunol.* 1994;152(4):1821–9. 8120391

[62] Kotenko SV Krause CD Izotova LS: Identification and functional characterization of a second chain of the interleukin-10 receptor complex. *EMBO J.* 1997;16(19):5894–903. 10.1093/emboj/16.19.5894 9312047 PMC1170220

[63] Spencer SD Di Marco F Hooley J: The orphan receptor CRF2-4 is an essential subunit of the interleukin 10 receptor. *J Exp Med.* 1998;187(4):571–8. 10.1084/jem.187.4.571 9463407 PMC2212143

[64] Donnelly RP Dickensheets H Finbloom DS: The interleukin-10 signal transduction pathway and regulation of gene expression in mononuclear phagocytes. *J Interferon Cytokine Res.* 1999;19(6):563–73. 10.1089/107999099313695 10433356

[65] Wehinger J Gouilleux F Groner B: IL-10 induces DNA binding activity of three STAT proteins (Stat1, Stat3, and Stat5) and their distinct combinatorial assembly in the promoters of selected genes. *FEBS Lett.* 1996;394(3):365–70. 10.1016/0014-5793(96)00990-8 8830676

[66] Weber-Nordt RM Riley JK Greenlund AC: Stat3 recruitment by two distinct ligand-induced, tyrosine-phosphorylated docking sites in the interleukin-10 receptor intracellular domain. *J Biol Chem.* 1996;271(44):27954–61. 10.1074/jbc.271.44.27954 8910398

[67] Riley JK Takeda K Akira S: Interleukin-10 receptor signaling through the JAK-STAT pathway. Requirement for two distinct receptor-derived signals for anti-inflammatory action. *J Biol Chem.* 1999;274(23):16513–21. 10.1074/jbc.274.23.16513 10347215

[68] Roers A Siewe L Strittmatter E: T cell-specific inactivation of the interleukin 10 gene in mice results in enhanced T cell responses but normal innate responses to lipopolysaccharide or skin irritation. *J Exp Med.* 2004;200(10):1289–97. 10.1084/jem.20041789 15534372 PMC2211912

[69] Medzhitov R: TLR-mediated innate immune recognition. *Semin Immunol.* 2007;19(1):1–2. 10.1016/j.smim.2007.02.001 22228983 PMC3252746

[70] Iwasaki A Medzhitov R: Regulation of adaptive immunity by the innate immune system. *Science.* 2010;327(5963):291–5. 10.1126/science.1183021 20075244 PMC3645875

[71] Brightbill HD Plevy SE Modlin RL: A prominent role for Sp1 during lipopolysaccharide-mediated induction of the IL-10 promoter in macrophages. *J Immunol.* 2000;164(4):1940–51. 10.4049/jimmunol.164.4.1940 10657644

[72] Liu YW Tseng HP Chen LC: Functional cooperation of simian virus 40 promoter factor 1 and CCAAT/enhancer-binding protein beta and delta in lipopolysaccharide-induced gene activation of IL-10 in mouse macrophages. *J Immunol.* 2003;171(2):821–8. 10.4049/jimmunol.171.2.821 12847250

[73] Cao S Liu J Song L: The protooncogene c-Maf is an essential transcription factor for IL-10 gene expression in macrophages. *J Immunol.* 2005;174(6):3484–92. 10.4049/jimmunol.174.6.3484 15749884 PMC2955976

[74] Liu YW Chen CC Tseng HP: Lipopolysaccharide-induced transcriptional activation of interleukin-10 is mediated by MAPK- and NF-kappaB-induced CCAAT/enhancer-binding protein delta in mouse macrophages. *Cell Signal.* 2006;18(9):1492–500. 10.1016/j.cellsig.2005.12.001 16413748

[75] Ananieva O Darragh J Johansen C: The kinases MSK1 and MSK2 act as negative regulators of Toll-like receptor signaling. *Nat Immunol.* 2008;9(9):1028–36. 10.1038/ni.1644 18690222

[76] MacKenzie KF Clark K Naqvi S: PGE _2_ induces macrophage IL-10 production and a regulatory-like phenotype via a protein kinase A-SIK-CRTC3 pathway. *J Immunol.* 2013;190(2):565–77. 10.4049/jimmunol.1202462 23241891 PMC3620524

[77] Kim SH Serezani CH Okunishi K: Distinct protein kinase A anchoring proteins direct prostaglandin E _2_ modulation of Toll-like receptor signaling in alveolar macrophages. *J Biol Chem.* 2011;286(11):8875–83. 10.1074/jbc.M110.187815 21247892 PMC3058957

[78] Pattison MJ MacKenzie KF Arthur JS: Inhibition of JAKs in macrophages increases lipopolysaccharide-induced cytokine production by blocking IL-10-mediated feedback. *J Immunol.* 2012;189(6):2784–92. 10.4049/jimmunol.1200310 22904308 PMC3443740

[79] Sayi A Kohler E Toller IM: TLR-2-activated B cells suppress *Helicobacter*-induced preneoplastic gastric immunopathology by inducing T regulatory-1 cells. *J Immunol.* 2011;186(2):878–90. 10.4049/jimmunol.1002269 21149607

[80] Agrawal S Gupta S: TLR1/2, TLR7, and TLR9 signals directly activate human peripheral blood naive and memory B cell subsets to produce cytokines, chemokines, and hematopoietic growth factors. *J Clin Immunol.* 2011;31(1):89–98. 10.1007/s10875-010-9456-8 20821041 PMC3064903

[81] Sun CM Deriaud E Leclerc C: Upon TLR9 signaling, CD5 ^+^ B cells control the IL-12-dependent Th1-priming capacity of neonatal DCs. *Immunity.* 2005;22(4):467–77. 10.1016/j.immuni.2005.02.008 15845451

[82] Wagner M Poeck H Jahrsdoerfer B: IL-12p70-dependent Th1 induction by human B cells requires combined activation with CD40 ligand and CpG DNA. *J Immunol.* 2004;172(2):954–63. 10.4049/jimmunol.172.2.954 14707068

[83] Schmidt NW Thieu VT Mann BA: Bruton's tyrosine kinase is required for TLR-induced IL-10 production. *J Immunol.* 2006;177(10):7203–10. 10.4049/jimmunol.177.10.7203 17082638

[84] Saraiva M O'Garra A: The regulation of IL-10 production by immune cells. *Nat Rev Immunol.* 2010;10(3):170–81. 10.1038/nri2711 20154735

[85] MacKenzie KF Pattison MJ Arthur JS: Transcriptional regulation of IL-10 and its cell-specific role *in vivo*. *Crit Rev Immunol.* 2014;34(4):315–45. 10.1615/CritRevImmunol.2014010694 24941159

[86] Voll RE Herrmann M Roth EA: Immunosuppressive effects of apoptotic cells. *Nature.* 1997;390(6658):350–1. 10.1038/37022 9389474

[87] D'Andrea A Aste-Amezaga M Valiante NM: Interleukin 10 (IL-10) inhibits human lymphocyte interferon gamma-production by suppressing natural killer cell stimulatory factor/IL-12 synthesis in accessory cells. *J Exp Med.* 1993;178(3):1041–8. 10.1084/jem.178.3.1041 8102388 PMC2191152

[88] Aste-Amezaga M Ma X Sartori A: Molecular mechanisms of the induction of IL-12 and its inhibition by IL-10. *J Immunol.* 1998;160(12):5936–44. 9637507

[89] Smith AM Qualls JE O'Brien K: A distal enhancer in *Il12b* is the target of transcriptional repression by the STAT3 pathway and requires the basic leucine zipper (B-ZIP) protein NFIL3. *J Biol Chem.* 2011;286(26):23582–90. 10.1074/jbc.M111.249235 21566115 PMC3123121

[90] Kobayashi T Matsuoka K Sheikh SZ: IL-10 regulates *Il12b* expression via histone deacetylation: implications for intestinal macrophage homeostasis. *J Immunol.* 2012;189(4):1792–9. 10.4049/jimmunol.1200042 22786766 PMC3411910

[91] Cao S Liu J Chesi M: Differential regulation of IL-12 and IL-10 gene expression in macrophages by the basic leucine zipper transcription factor c-Maf fibrosarcoma. *J Immunol.* 2002;169(10):5715–25. 10.4049/jimmunol.169.10.5715 12421951

[92] Krausgruber T Blazek K Smallie T: IRF5 promotes inflammatory macrophage polarization and T _H_1-T _H_17 responses. *Nat Immunol.* 2011;12(3):231–8. 10.1038/ni.1990 21240265

[93] Colombo MP Trinchieri G: Interleukin-12 in anti-tumor immunity and immunotherapy. *Cytokine Growth Factor Rev.* 2002;13(2):155–68. 10.1016/S1359-6101(01)00032-6 11900991

[94] Shi X Cao S Mitsuhashi M: Genome-wide analysis of molecular changes in IL-12-induced control of mammary carcinoma via IFN-gamma-independent mechanisms. *J Immunol.* 2004;172(7):4111–22. 10.4049/jimmunol.172.7.4111 15034023 PMC2956987

[95] Simpson-Abelson MR Purohit VS Pang WM: IL-12 delivered intratumorally by multilamellar liposomes reactivates memory T cells in human tumor microenvironments. *Clin Immunol.* 2009;132(1):71–82. 10.1016/j.clim.2009.03.516 19395317 PMC2693480

[96] Helms MW Prescher JA Cao YA: IL-12 enhances efficacy and shortens enrichment time in cytokine-induced killer cell immunotherapy. *Cancer Immunol Immunother.* 2010;59(9):1325–34. 10.1007/s00262-010-0860-y 20532883 PMC4922493

[97] Marzano AV Gasparini G Caputo R: Subcutaneous sarcoidosis following hypophysectomy for pituitary microadenoma inducing Cushing's disease. *Int J Dermatol.* 1998;37(10):798. 10.1046/j.1365-4362.1998.00525.x 9802695

[98] Denies S Cicchelero L Van Audenhove I: Combination of interleukin-12 gene therapy, metronomic cyclophosphamide and DNA cancer vaccination directs all arms of the immune system towards tumor eradication. *J Control Release.* 2014;187:175–82. 10.1016/j.jconrel.2014.05.045 24887014

[99] Vom Berg J Vrohlings M Haller S: Intratumoral IL-12 combined with CTLA-4 blockade elicits T cell-mediated glioma rejection. *J Exp Med.* 2013;210(13):2803–11. 10.1084/jem.20130678 24277150 PMC3865478

[100] Vogt A Sievers E Lukacs-Kornek V: Improving immunotherapy of hepatocellular carcinoma (HCC) using dendritic cells (DC) engineered to express IL-12 *in vivo*. *Liver Int.* 2014;34(3):447–61. 10.1111/liv.12284 23998316

[101] Miguel A Herrero MJ Sendra L: Comparative antitumor effect among GM-CSF, IL-12 and GM-CSF+IL-12 genetically modified tumor cell vaccines. *Cancer Gene Ther.* 2013;20(10):576–81. 10.1038/cgt.2013.54 23969885

[102] He H Fan P Yin T: Local delivery of recombinant adenovirus expressing hepatitis B virus X protein and interleukin-12 results in antitumor effects via inhibition of hepatoma cell growth and intervention of tumor microenvironment. *Int J Mol Med.* 2012;30(3):599–605. 10.3892/ijmm.2012.1027 22710287

[103] Malvicini M Ingolotti M Piccioni F: Reversal of gastrointestinal carcinoma-induced immunosuppression and induction of antitumoural immunity by a combination of cyclophosphamide and gene transfer of IL-12. *Mol Oncol.* 2011;5(3):242–55. 10.1016/j.molonc.2011.03.007 21515097 PMC5528288

[104] Kerkar SP Muranski P Kaiser A: Tumor-specific CD8 ^+^ T cells expressing interleukin-12 eradicate established cancers in lymphodepleted hosts. *Cancer Res.* 2010;70(17):6725–34. 10.1158/0008-5472.CAN-10-0735 20647327 PMC2935308

[105] Malvicini M Rizzo M Alaniz L: A novel synergistic combination of cyclophosphamide and gene transfer of interleukin-12 eradicates colorectal carcinoma in mice. *Clin Cancer Res.* 2009;15(23):7256–65. 10.1158/1078-0432.CCR-09-1861 19920110

[106] Yuzhalin AE Kutikhin AG: Interleukin-12: clinical usage and molecular markers of cancer susceptibility. *Growth Factors.* 2012;30(3):176–91. 10.3109/08977194.2012.678843 22515181

[107] Ramos CA Savoldo B Dotti G: CD19-CAR trials. *Cancer J.* 2014;20(2):112–8. 10.1097/PPO.0000000000000031 24667955 PMC3979594

[108] Chmielewski M Kopecky C Hombach AA: IL-12 release by engineered T cells expressing chimeric antigen receptors can effectively Muster an antigen-independent macrophage response on tumor cells that have shut down tumor antigen expression. *Cancer Res.* 2011;71(17):5697–706. 10.1158/0008-5472.CAN-11-0103 21742772

[109] Zhang L Kerkar SP Yu Z: Improving adoptive T cell therapy by targeting and controlling IL-12 expression to the tumor environment. *Mol Ther.* 2011;19(4):751–9. 10.1038/mt.2010.313 21285960 PMC3070103

[110] Zhang L Feldman SA Zheng Z: Evaluation of γ-retroviral vectors that mediate the inducible expression of IL-12 for clinical application. *J Immunother.* 2012;35(5):430–9. 10.1097/CJI.0b013e31825898e8 22576348 PMC3358728

[111] King IL Segal BM: Cutting edge: IL-12 induces CD4 ^+^CD25 ^-^ T cell activation in the presence of T regulatory cells. *J Immunol.* 2005;175(2):641–5. 10.4049/jimmunol.175.2.641 16002658

[112] Oldenhove G Bouladoux N Wohlfert EA: Decrease of Foxp3 ^+^ Treg cell number and acquisition of effector cell phenotype during lethal infection. *Immunity.* 2009;31(5):772–86. 10.1016/j.immuni.2009.10.001 19896394 PMC2814877

[113] Feng T Cao AT Weaver CT: Interleukin-12 converts Foxp3 ^+^ regulatory T cells to interferon-γ-producing Foxp3 ^+^ T cells that inhibit colitis. *Gastroenterology.* 2011;140(7):2031–43. 10.1053/j.gastro.2011.03.009 21419767 PMC3109200

[114] Zhao J Zhao J Perlman S: Differential effects of IL-12 on Tregs and non-Treg T cells: roles of IFN-γ, IL-2 and IL-2R. *PLoS One.* 2012;7(9):e46241. 10.1371/journal.pone.0046241 23029447 PMC3459844

[115] Kerkar SP Goldszmid RS Muranski P: IL-12 triggers a programmatic change in dysfunctional myeloid-derived cells within mouse tumors. *J Clin Invest.* 2011;121(12):4746–57. 10.1172/JCI58814 22056381 PMC3226001

[116] Broderick L Brooks SP Takita H: IL-12 reverses anergy to T cell receptor triggering in human lung tumor-associated memory T cells. *Clin Immunol.* 2006;118(2–3):159–69. 10.1016/j.clim.2005.09.008 16271513

[117] Kilinc MO Aulakh KS Nair RE: Reversing tumor immune suppression with intratumoral IL-12: activation of tumor-associated T effector/memory cells, induction of T suppressor apoptosis, and infiltration of CD8 ^+^ T effectors. *J Immunol.* 2006;177(10):6962–73. 10.4049/jimmunol.177.10.6962 17082611

[118] Watkins SK Egilmez NK Suttles J: IL-12 rapidly alters the functional profile of tumor-associated and tumor-infiltrating macrophages *in vitro* and *in vivo*. *J Immunol.* 2007;178(3):1357–62. 10.4049/jimmunol.178.3.1357 17237382

[119] Curtsinger JM Lins DC Mescher MF: Signal 3 determines tolerance versus full activation of naive CD8 T cells: dissociating proliferation and development of effector function. *J Exp Med.* 2003;197(9):1141–51. 10.1084/jem.20021910 12732656 PMC2193970

[120] O'Garra A Barrat FJ Castro AG: Strategies for use of IL-10 or its antagonists in human disease. *Immunol Rev.* 2008;223(1):114–31. 10.1111/j.1600-065X.2008.00635.x 18613832

[121] Baglaenko Y Manion KP Chang NH: Suppression of autoimmunity by CD5 ^+^ IL-10-producing B cells in lupus-prone mice. *Genes Immun.* 2015;16(5):311–20. 10.1038/gene.2015.17 25973757

[122] Frenkel D Huang Z Maron R: Neuroprotection by IL-10-producing MOG CD4+ T cells following ischemic stroke. *J Neurol Sci.* 2005;233(1–2):125–32. 10.1016/j.jns.2005.03.022 15894335

[123] Gangi E Vasu C Cheatem D: IL-10-producing CD4 ^+^CD25 ^+^ regulatory T cells play a critical role in granulocyte-macrophage colony-stimulating factor-induced suppression of experimental autoimmune thyroiditis. *J Immunol.* 2005;174(11):7006–13. 10.4049/jimmunol.174.11.7006 15905543

[124] Lavasani S Dzhambazov B Nouri M: A novel probiotic mixture exerts a therapeutic effect on experimental autoimmune encephalomyelitis mediated by IL-10 producing regulatory T cells. *PLoS One.* 2010;5(2):e9009. 10.1371/journal.pone.0009009 20126401 PMC2814855

[125] Matsushita T Yanaba K Bouaziz JD: Regulatory B cells inhibit EAE initiation in mice while other B cells promote disease progression. *J Clin Invest.* 2008;118(10):3420–30. 10.1172/JCI36030 18802481 PMC2542851

[126] Yang M Deng J Liu Y: IL-10-producing regulatory B10 cells ameliorate collagen-induced arthritis via suppressing Th17 cell generation. *Am J Pathol.* 2012;180(6):2375–85. 10.1016/j.ajpath.2012.03.010 22538089

[127] Yang S Li W Liu W: IL-10 gene modified dendritic cells induced antigen-specific tolerance in experimental autoimmune myocarditis. *Clin Immunol.* 2006;121(1):63–73. 10.1016/j.clim.2006.06.009 16904381

[128] Brand S: Crohn's disease: Th1, Th17 or both? The change of a paradigm: new immunological and genetic insights implicate Th17 cells in the pathogenesis of Crohn's disease. *Gut.* 2009;58(8):1152–67. 10.1136/gut.2008.163667 19592695

[129] Noguchi E Homma Y Kang X: A Crohn's disease-associated *NOD2* mutation suppresses transcription of human *IL10* by inhibiting activity of the nuclear ribonucleoprotein hnRNP-A1. *Nat Immunol.* 2009;10(5):471–9. 10.1038/ni.1722 19349988 PMC2928218

[130] Wang ZE Zheng S Corry DB: Interferon gamma-independent effects of interleukin 12 administered during acute or established infection due to Leishmania major. *Proc Natl Acad Sci U S A.* 1994;91(26):12932–6. 10.1073/pnas.91.26.12932 7809149 PMC45554

[131] Taylor AP Murray HW: Intracellular antimicrobial activity in the absence of interferon-gamma: effect of interleukin-12 in experimental visceral leishmaniasis in interferon-gamma gene-disrupted mice. *J Exp Med.* 1997;185(7):1231–9. 10.1084/jem.185.7.1231 9104810 PMC2196266

[132] Zilocchi C Stoppacciaro A Chiodoni C: Interferon gamma-independent rejection of interleukin 12-transduced carcinoma cells requires CD4 ^+^ T cells and Granulocyte/Macrophage colony-stimulating factor. *J Exp Med.* 1998;188(1):133–43. 10.1084/jem.188.1.133 9653090 PMC2525540

[133] Roilides E Tsaparidou S Kadiltsoglou I: Interleukin-12 enhances antifungal activity of human mononuclear phagocytes against *Aspergillus fumigatus*: implications for a gamma interferon-independent pathway. *Infect Immun.* 1999;67(6):3047–50. 10338518 10.1128/iai.67.6.3047-3050.1999 PMC96619

[134] Hafner M Falk W Echtenacher B: Interleukin-12 activates NK cells for IFN-gamma-dependent and NKT cells for IFN-gamma-independent antimetastatic activity. *Eur Cytokine Netw.* 1999;10(4):541–8. 10586121

[135] Afanasyeva M Wang Y Kaya Z: Interleukin-12 receptor/STAT4 signaling is required for the development of autoimmune myocarditis in mice by an interferon-gamma-independent pathway. *Circulation.* 2001;104(25):3145–51. 10.1161/hc5001.100629 11748115

[136] Shi X Liu J Xiang Z: Gene expression analysis in Interleukin-12-induced suppression of mouse mammary carcinoma. *Int J Cancer.* 2004;110(4):570–8. 10.1002/ijc.20145 15122590 PMC2957898

[137] Meyaard L Hovenkamp E Otto SA: IL-12-induced IL-10 production by human T cells as a negative feedback for IL-12-induced immune responses. *J Immunol.* 1996;156(8):2776–82. 8609396

[138] Portielje JE Lamers CH Kruit WH: Repeated administrations of interleukin (IL)-12 are associated with persistently elevated plasma levels of IL-10 and declining IFN-gamma, tumor necrosis factor-alpha, IL-6, and IL-8 responses. *Clin Cancer Res.* 2003;9(1):76–83. 12538454

[139] Gerosa F Paganin C Peritt D: Interleukin-12 primes human CD4 and CD8 T cell clones for high production of both interferon-gamma and interleukin-10. *J Exp Med.* 1996;183(6):2559–69. 10.1084/jem.183.6.2559 8676077 PMC2192598

[140] Wysocka M Robertson S Riemann H: IL-12 suppression during experimental endotoxin tolerance: dendritic cell loss and macrophage hyporesponsiveness. *J Immunol.* 2001;166(12):7504–13. 10.4049/jimmunol.166.12.7504 11390504

[141] Foster SL Hargreaves DC Medzhitov R: Gene-specific control of inflammation by TLR-induced chromatin modifications. *Nature.* 2007;447(7147):972–8. 10.1038/nature05836 17538624

[142] He R Shepard LW Chen J: Serum amyloid A is an endogenous ligand that differentially induces IL-12 and IL-23. *J Immunol.* 2006;177(6):4072–9. 10.4049/jimmunol.177.6.4072 16951371

[143] Waibler Z Kalinke U Will J: TLR-ligand stimulated interleukin-23 subunit expression and assembly is regulated differentially in murine plasmacytoid and myeloid dendritic cells. *Mol Immunol.* 2007;44(7):1483–9. 10.1016/j.molimm.2006.09.001 17052755

[144] Gillessen S Carvajal D Ling P: Mouse interleukin-12 (IL-12) p40 homodimer: a potent IL-12 antagonist. *Eur J Immunol.* 1995;25(1):200–6. 10.1002/eji.1830250133 7843232

[145] Ling P Gately MK Gubler U: Human IL-12 p40 homodimer binds to the IL-12 receptor but does not mediate biologic activity. *J Immunol.* 1995;154(1):116–27. 7527811

[146] Heckel MC Wolfson A Slachta CA: Human breast tumor cells express IL-10 and IL-12p40 transcripts and proteins, but do not produce IL-12p70. *Cell Immunol.* 2011;266(2):143–53. 10.1016/j.cellimm.2010.09.010 21055733

[147] Heinzel FP Hujer AM Ahmed FN: *In vivo* production and function of IL-12 p40 homodimers. *J Immunol.* 1997;158(9):4381–8. 9127002

[148] Mattner F Ozmen L Podlaski FJ: Treatment with homodimeric interleukin-12 (IL-12) p40 protects mice from IL-12-dependent shock but not from tumor necrosis factor alpha-dependent shock. *Infect Immun.* 1997;65(11):4734–7. 9353058 10.1128/iai.65.11.4734-4737.1997 PMC175679

[149] Piccotti JR Chan SY Li K: Differential effects of IL-12 receptor blockade with IL-12 p40 homodimer on the induction of CD4 ^+^ and CD8 ^+^ IFN-gamma-producing cells. *J Immunol.* 1997;158(2):643–8. 8992979

[150] Pegram HJ Lee JC Hayman EG: Tumor-targeted T cells modified to secrete IL-12 eradicate systemic tumors without need for prior conditioning. *Blood.* 2012;119(18):4133–41. 10.1182/blood-2011-12-400044 22354001 PMC3359735

[151] Koneru M Purdon TJ Spriggs D: IL-12 secreting tumor-targeted chimeric antigen receptor T cells eradicate ovarian tumors *in vivo*. *Oncoimmunology.* 2015;4(3):e994446. 10.4161/2162402X.2014.994446 25949921 PMC4404840

